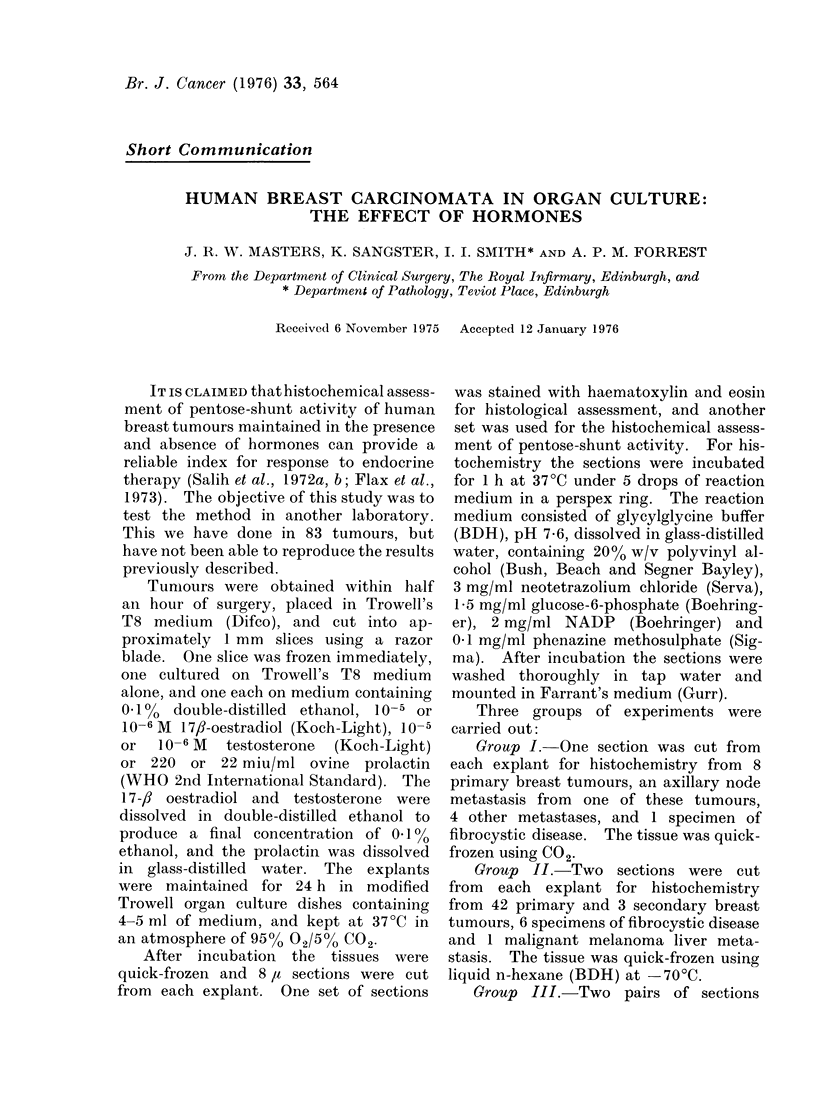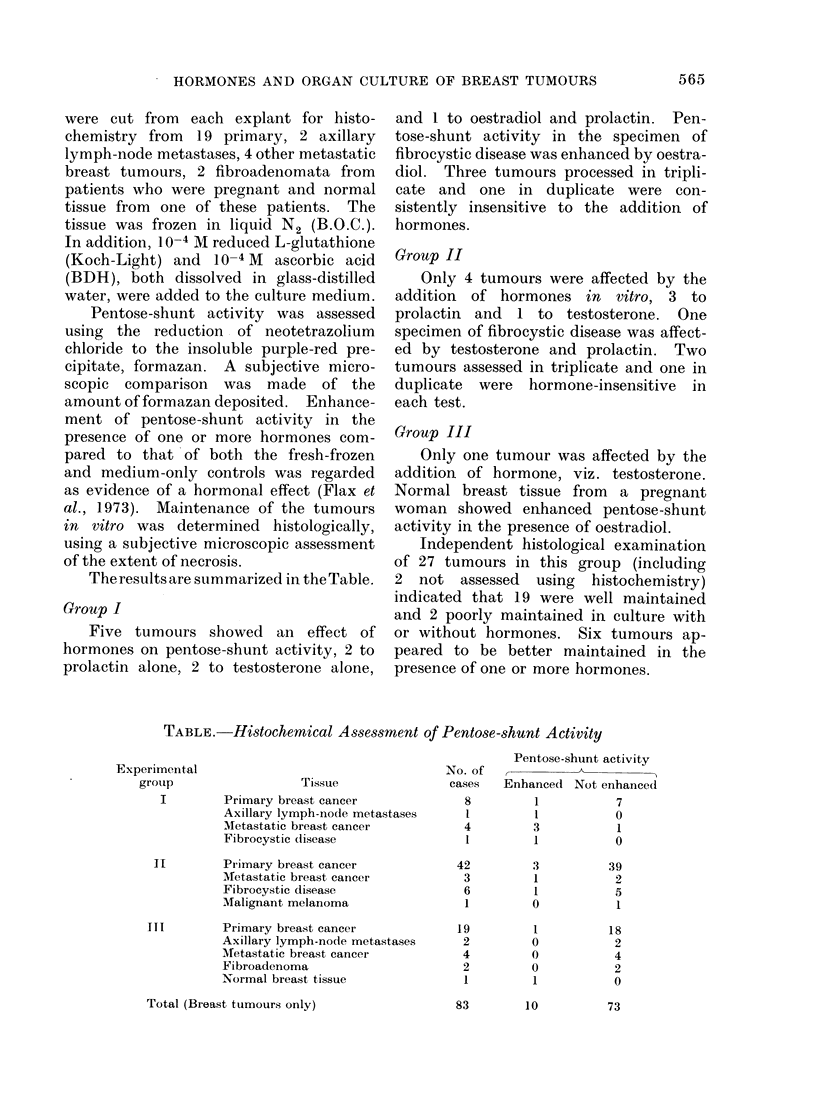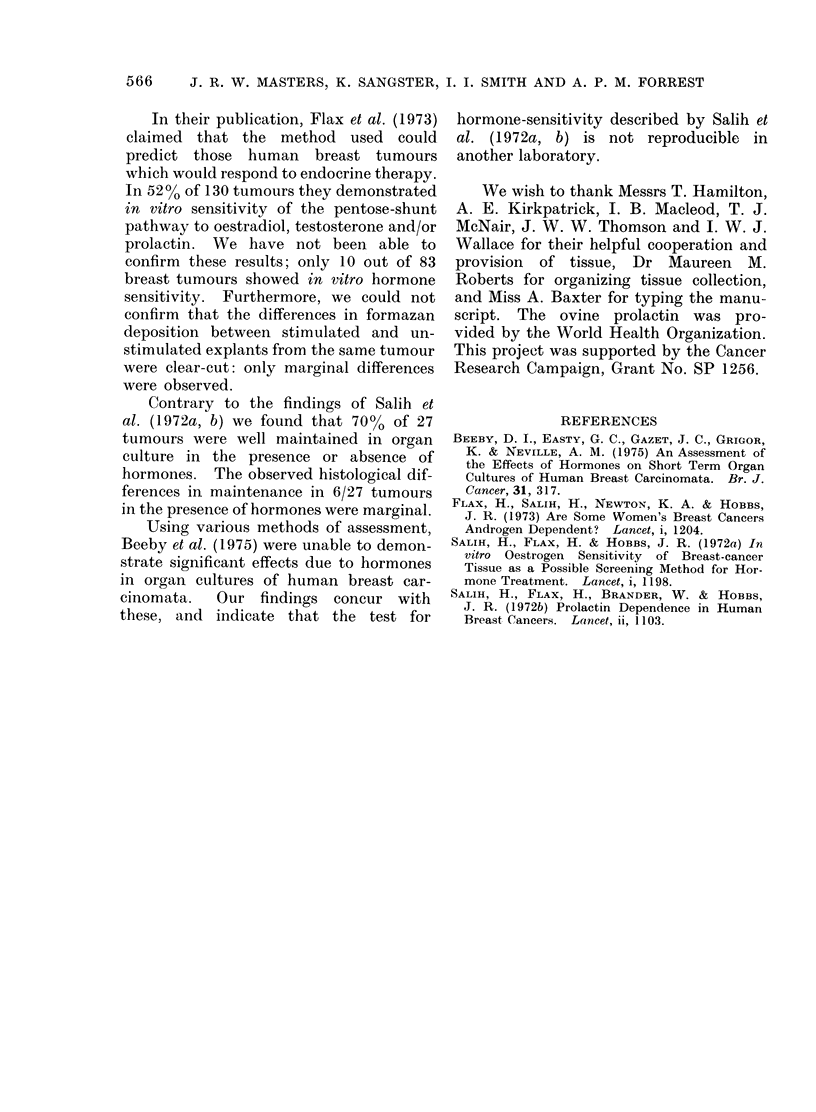# Human breast carcinomata in organ culture: the effect of hormones.

**DOI:** 10.1038/bjc.1976.90

**Published:** 1976-05

**Authors:** J. R. Masters, K. Sangster, I. I. Smith, A. P. Forrest


*Br. J. Cancer* (1976) **33**, 564

***Short Communication***

### HUMAN BREAST CARCINOMATA IN ORGAN CULTURE: THE EFFECT OF HORMONES

J. R. W. MASTERS, K. SANGSTER, I. I. SMITH* AND A. P. M. FORREST

*From the Department of Clinical Surgery, The Royal Infirmary, Edinburgh, and * Department of Pathology, Teviot Place, Edinburgh*

Received 6 November 1975 Accepted 12 January 1976

IT IS CLAIMED that histochemical assessment of pentose-shunt activity of human breast tumours maintained in the presence and absence of hormones can provide a reliable index for response to endocrine therapy (Salih *et al.*, 1972*a*, *b*; Flax *et al.*, 1973). The objective of this study was to test the method in another laboratory. This we have done in 83 tumours, but have not been able to reproduce the results previously described.

Tumours were obtained within half an hour of surgery, placed in Trowell's T8 medium (Difco), and cut into approximately 1 mm slices using a razor blade. One slice was frozen immediately, one cultured on Trowell's T8 medium alone, and one each on medium containing 0·1% double-distilled ethanol, $10^{-5}$ or $10^{-6}$ M 17$\beta$-oestradiol (Koch-Light), $10^{-5}$ or $10^{-6}$ M testosterone (Koch-Light) or 220 or 22 miu/ml ovine prolactin (WHO 2nd International Standard). The 17-$\beta$ oestradiol and testosterone were dissolved in double-distilled ethanol to produce a final concentration of 0·1% ethanol, and the prolactin was dissolved in glass-distilled water. The explants were maintained for 24 h in modified Trowell organ culture dishes containing 4–5 ml of medium, and kept at 37°C in an atmosphere of 95% $O_2$/5% $CO_2$.

After incubation the tissues were quick-frozen and 8 $\mu$ sections were cut from each explant. One set of sections was stained with haematoxylin and eosin for histological assessment, and another set was used for the histochemical assessment of pentose-shunt activity. For histochemistry the sections were incubated for 1 h at 37°C under 5 drops of reaction medium in a perspex ring. The reaction medium consisted of glycylglycine buffer (BDH), pH 7·6, dissolved in glass-distilled water, containing 20% w/v polyvinyl alcohol (Bush, Beach and Segner Bayley), 3 mg/ml neotetrazolium chloride (Serva), 1·5 mg/ml glucose-6-phosphate (Boehringer), 2 mg/ml NADP (Boehringer) and 0·1 mg/ml phenazine methosulphate (Sigma). After incubation the sections were washed thoroughly in tap water and mounted in Farrant's medium (Gurr).

Three groups of experiments were carried out:

*Group I.*—One section was cut from each explant for histochemistry from 8 primary breast tumours, an axillary node metastasis from one of these tumours, 4 other metastases, and 1 specimen of fibrocystic disease. The tissue was quick-frozen using $CO_2$.

*Group II.*—Two sections were cut from each explant for histochemistry from 42 primary and 3 secondary breast tumours, 6 specimens of fibrocystic disease and 1 malignant melanoma liver metastasis. The tissue was quick-frozen using liquid n-hexane (BDH) at −70°C.

*Group III.*—Two pairs of sections were cut from each explant for histochemistry from 19 primary, 2 axillary lymph-node metastases, 4 other metastatic breast tumours, 2 fibroadenomata from patients who were pregnant and normal tissue from one of these patients. The tissue was frozen in liquid $N_2$ (B.O.C.). In addition, $10^{-4}$ M reduced L-glutathione (Koch-Light) and $10^{-4}$ M ascorbic acid (BDH), both dissolved in glass-distilled water, were added to the culture medium.

Pentose-shunt activity was assessed using the reduction of neotetrazolium chloride to the insoluble purple-red precipitate, formazan. A subjective microscopic comparison was made of the amount of formazan deposited. Enhancement of pentose-shunt activity in the presence of one or more hormones compared to that of both the fresh-frozen and medium-only controls was regarded as evidence of a hormonal effect (Flax *et al.*, 1973). Maintenance of the tumours *in vitro* was determined histologically, using a subjective microscopic assessment of the extent of necrosis.

The results are summarized in the Table.

*Group I*

Five tumours showed an effect of hormones on pentose-shunt activity, 2 to prolactin alone, 2 to testosterone alone, and 1 to oestradiol and prolactin. Pentose-shunt activity in the specimen of fibrocystic disease was enhanced by oestradiol. Three tumours processed in triplicate and one in duplicate were consistently insensitive to the addition of hormones.

*Group II*

Only 4 tumours were affected by the addition of hormones *in vitro*, 3 to prolactin and 1 to testosterone. One specimen of fibrocystic disease was affected by testosterone and prolactin. Two tumours assessed in triplicate and one in duplicate were hormone-insensitive in each test.

*Group III*

Only one tumour was affected by the addition of hormone, viz. testosterone. Normal breast tissue from a pregnant woman showed enhanced pentose-shunt activity in the presence of oestradiol.

Independent histological examination of 27 tumours in this group (including 2 not assessed using histochemistry) indicated that 19 were well maintained and 2 poorly maintained in culture with or without hormones. Six tumours appeared to be better maintained in the presence of one or more hormones.

TABLE.—*Histochemical Assessment of Pentose-shunt Activity*

| Experimental group | Tissue | No. of cases | Pentose-shunt activity | |
|---|---|---|---|---|
| | | | Enhanced | Not enhanced |
| I | Primary breast cancer | 8 | 1 | 7 |
| | Axillary lymph-node metastases | 1 | 1 | 0 |
| | Metastatic breast cancer | 4 | 3 | 1 |
| | Fibrocystic disease | 1 | 1 | 0 |
| II | Primary breast cancer | 42 | 3 | 39 |
| | Metastatic breast cancer | 3 | 1 | 2 |
| | Fibrocystic disease | 6 | 1 | 5 |
| | Malignant melanoma | 1 | 0 | 1 |
| III | Primary breast cancer | 19 | 1 | 18 |
| | Axillary lymph-node metastases | 2 | 0 | 2 |
| | Metastatic breast cancer | 4 | 0 | 4 |
| | Fibroadenoma | 2 | 0 | 2 |
| | Normal breast tissue | 1 | 1 | 0 |
| Total (Breast tumours only) | | 83 | 10 | 73 |

In their publication, Flax *et al.* (1973) claimed that the method used could predict those human breast tumours which would respond to endocrine therapy. In 52% of 130 tumours they demonstrated *in vitro* sensitivity of the pentose-shunt pathway to oestradiol, testosterone and/or prolactin. We have not been able to confirm these results; only 10 out of 83 breast tumours showed *in vitro* hormone sensitivity. Furthermore, we could not confirm that the differences in formazan deposition between stimulated and unstimulated explants from the same tumour were clear-cut: only marginal differences were observed.

Contrary to the findings of Salih *et al.* (1972*a*, *b*) we found that 70% of 27 tumours were well maintained in organ culture in the presence or absence of hormones. The observed histological differences in maintenance in 6/27 tumours in the presence of hormones were marginal.

Using various methods of assessment, Beeby *et al.* (1975) were unable to demonstrate significant effects due to hormones in organ cultures of human breast carcinomata. Our findings concur with these, and indicate that the test for hormone-sensitivity described by Salih *et al.* (1972*a*, *b*) is not reproducible in another laboratory.

We wish to thank Messrs T. Hamilton, A. E. Kirkpatrick, I. B. Macleod, T. J. McNair, J. W. W. Thomson and I. W. J. Wallace for their helpful cooperation and provision of tissue, Dr Maureen M. Roberts for organizing tissue collection, and Miss A. Baxter for typing the manuscript. The ovine prolactin was provided by the World Health Organization. This project was supported by the Cancer Research Campaign, Grant No. SP 1256.

### REFERENCES

BEEBY, D. I., EASTY, G. C., GAZET, J. C., GRIGOR, K. & NEVILLE, A. M. (1975) An Assessment of the Effects of Hormones on Short Term Organ Cultures of Human Breast Carcinomata. *Br. J. Cancer*, **31**, 317.

FLAX, H., SALIH, H., NEWTON, K. A. & HOBBS, J. R. (1973) Are Some Women's Breast Cancers Androgen Dependent? *Lancet*, i, 1204.

SALIH, H., FLAX, H. & HOBBS, J. R. (1972*a*) *In vitro* Oestrogen Sensitivity of Breast-cancer Tissue as a Possible Screening Method for Hormone Treatment. *Lancet*, i, 1198.

SALIH, H., FLAX, H., BRANDER, W. & HOBBS, J. R. (1972*b*) Prolactin Dependence in Human Breast Cancers. *Lancet*, ii, 1103.